# Longitudinal Assessment of Dental Erosion in a Romanian Cohort of Young Adults: A Ten-Year Follow-Up Pilot Study

**DOI:** 10.3390/dj13070302

**Published:** 2025-07-03

**Authors:** Andrea Bors, Felicia Gabriela Beresescu, Melinda Szekely

**Affiliations:** Faculty of Dentistry, George Emil Palade University of Medicine, Pharmacy, Science, and Technology, 38 Gheorghe Marinescu Street, 540142 Târgu Mureș, Romania; melinda.szekely@umfst.ro

**Keywords:** BEWE index, diagnostic, digital dentistry, enamel wear, gender differences, intraoral scanning, lesions, longitudinal study, preventive dentistry, Romanian cohort

## Abstract

**Background:** Dental erosion is the irreversible loss of tooth structure from acid exposure. Its prevalence is rising globally, making it an important oral health concern. However, longitudinal data from Eastern Europe are scarce, especially in Romania. This pilot study aimed to assess the 10-year incidence of dental erosion in Romanian young adults and to compare clinical index scoring with digital scanning. **Methods:** A 10-year prospective study followed 540 Romanian adults (aged 18–30) selected with no erosive lesions at baseline (Basic Erosive Wear Examination BEWE = 0). Erosive wear was assessed at the 10-year follow-up using BEWE, with 40 participants also undergoing digital intraoral scanning to measure enamel loss (μm). Gender differences were analyzed. Chi-square tests, relative risk, and correlation analyses were performed. **Results:** After 10 years, 23.2% of participants developed dental erosion. Males exhibited a higher incidence than females (29.9% vs. 17.2%; RR = 1.74, *p* < 0.001). Among the scanned subset (*n* = 40), the mean enamel loss was 137 ± 79 µm, with greater wear on palatal vs. buccal surfaces (*p* = 0.002). BEWE scores were moderately correlated with digital enamel loss (*r* = 0.58, *p* < 0.001). **Conclusions:** Erosion progressed over time in this cohort, with males at higher risk. Digital scanning detected subtle enamel loss not captured by BEWE, indicating greater sensitivity to early changes. BEWE and digital methods provided complementary information; their combined use offers a more comprehensive assessment.

## 1. Introduction

Dental erosion, defined as the irreversible loss of dental hard tissue due to chemical processes not involving bacterial activity, has emerged as a growing oral health concern in the 21st century [1]. Unlike caries and periodontal disease, which are associated with bacterial biofilms, dental erosion progresses due to extrinsic or intrinsic chemical challenges acting directly on the tooth surface. These processes may go unnoticed until significant structural injury has occurred, leading to functional and esthetic complications [2].

The diagnosis of erosion may be clinically challenging due to its asymptomatic progression. Patients are often unaware of the condition until dentin becomes exposed, or the tooth surface undergoes visible morphological change. In many cases, early-stage lesions are missed during routine dental examinations, contributing to underdiagnosis and delayed intervention [3]. The importance of identifying erosion early is underscored by its irreversible nature and potential to affect the quality of life through hypersensitivity, enamel translucency loss, and restorative complications [4].

Erosive tooth wear (ETW) is a multifactorial condition encompassing the progressive and irreversible loss of dental hard tissue due to chemical, mechanical, or combined actions [1]. Dental erosion—defined as the chemical loss of enamel and dentin from acids not derived from bacteria—is one of the principal causes of ETW. While dental erosion specifically refers to the chemical process, ETW often includes attrition (tooth-to-tooth contact) and abrasion (external mechanical forces). These processes may interact synergistically, with acidic softening of enamel surfaces increasing susceptibility to mechanical wear. As such, assessing dental erosion within the broader context of ETW is crucial, particularly in young adults, where lifestyle factors such as dietary acids and parafunctional habits can accelerate the condition’s onset and progression. The current study focuses on the chemical component—dental erosion—while acknowledging its clinical overlap with other wear mechanisms [2].

Global evidence suggests that dental erosion is increasingly common among young adults [2]. Recent systematic reviews and meta-analyses estimate the worldwide prevalence of erosive tooth wear in youth to range from 25% to over 50%, depending on age group, region, and diagnostic criteria [5,6,7]. However, the incidence of dental erosion—defined as the number of new cases developing over a period of time—is rarely reported, particularly in prospective studies. While prevalence data reflect a snapshot of disease burden, incidence studies are crucial for understanding the dynamic nature of disease progression and for identifying emerging trends in specific populations [7].

Despite growing interest in erosion-related research globally, there is a notable lack of longitudinal studies in Eastern European populations [8,9,10,11,12]. Most research from this region, including Romania, remains cross-sectional and does not provide information on the natural course of erosive wear [13]. In particular, there is limited data on erosion development in young adults—a group often considered at the early stages of exposure and disease onset. Understanding erosion incidence in this population could provide important guidance for preventive strategies, early diagnosis, and targeted public health messaging [12].

Furthermore, little is known about how demographic variables such as gender may influence the development of erosive lesions over time. However, studies have suggested that males may be at a higher risk due to behavioral, anatomical, or physiological factors [12]; still, findings remain inconsistent and underexplored in Eastern European contexts [11].

The BEWE index was first introduced in 2008 by Bartlett et al. as a simplified, standardized scoring system designed to facilitate clinical and epidemiological assessments of erosive tooth wear [14]. Since then, it has become a cornerstone tool in both general dentistry and research for tracking the severity and progression of non-carious enamel loss. For clinical and research purposes, the Basic Erosive Wear Examination (BEWE) index has been widely adopted as a standardized method for assessing erosive tooth wear [8]. Its structure allows clinicians to classify severity by scoring the most affected surface in each sextant, generating a cumulative score that guides clinical risk assessment and monitoring over time. The BEWE has shown good intra- and inter-examiner reliability and has been recommended by the European consensus group on tooth wear as a suitable tool for use in both general practice and epidemiological research [15].

With advancements in digital dentistry, intraoral scanning technology has introduced novel possibilities for objective and reproducible measurement of enamel loss. By capturing high-resolution, three-dimensional surface data, intraoral scanners enable digital quantification of dental erosion over successive time points. This approach allows for precise monitoring of changes in enamel volume or surface contours, overcoming some limitations of visual indices and providing a valuable validation tool for clinical findings [16].

The present longitudinal study was conducted over a ten-year period in Târgu Mureș, Romania, with the following aims:

1. To assess the ten-year incidence of dental erosion in a cohort of healthy young adults using the BEWE index.
2. To investigate whether gender or other demographic factors are associated with the development of new erosive lesions.
3. To evaluate the sensitivity of the digital surface analysis in detecting early or localized erosive changes not captured by BEWE.
4. To validate clinical findings through 3D digital surface loss analysis using intraoral scanning and quantitative measurement of enamel wear.

## 2. Materials and Methods

### 2.1. Study Design and Ethical Approval

This prospective, longitudinal observational study was conducted over ten years (2014–2024) in Târgu Mureș, Romania. The study aimed to evaluate the incidence and progression of dental erosion in a cohort of healthy young adults The study was approved by the Institutional Ethics Committee of the University of Medicine and Pharmacy of Târgu Mureș (Approval No. 432/2014, approved on 15 June 2014) and written informed consent was obtained from all participants in accordance with the Declaration of Helsinki.

#### Participant Selection and Follow-Up

A total of 540 healthy adults aged 18–30 years were recruited in 2014 from university and community clinics. Inclusion required a full dentition (excluding third molars) and a BEWE score of 0 (no signs of erosion) at baseline. Exclusion criteria included:

- Diagnosed with gastroesophageal reflux disease (GERD) or eating disorders.
- Use of medications causing xerostomia.
- Fixed orthodontic appliances or extensive restorations affecting enamel integrity.
- At the 2024 follow-up, 517 participants (95.7%) were successfully re-examined; 23 participants were lost to follow-up.

**Figure 1:**
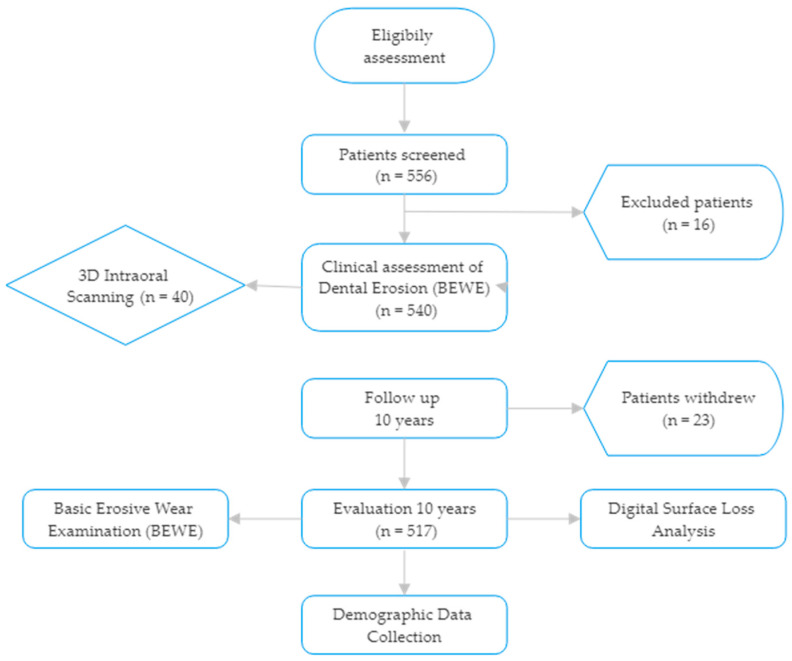
Study flowchart.

The flow of participants throughout the study is illustrated in Figure 1.

### 2.2. Clinical Assessment of Dental Erosion

#### 2.2.1. Basic Erosive Wear Examination (BEWE)

Dental erosion was assessed at baseline and follow-up using the BEWE index [15]. The dentition was divided into six sextants, and the most severely affected tooth surface in each sextant was scored as follows: 0: No erosion; 1: Initial loss of surface texture; 2: Distinct hard tissue loss < 50% of the surface area; and 3: Hard tissue loss ≥ 50% of the surface area.

A BEWE score ≥ 1 in any sextant at follow-up indicated dental erosion diagnosis. All clinical assessments were performed by two calibrated examiners, and inter-examiner reliability was high (Cohen’s kappa = 0.87).

To ensure diagnostic consistency, two experienced examiners were calibrated prior to the study through training sessions involving clinical photographs and direct patient assessments using the BEWE criteria. Calibration was validated using a standardized set of cases. Inter-examiner reliability was assessed at baseline and during follow-up, with Cohen’s kappa value calculated at 0.87, indicating strong agreement. Intra-examiner consistency was periodically re-verified to maintain scoring accuracy throughout the study period.

Impressions for each patient were recorded, and casts were poured and appropriately marked to be recognized during follow-up.

#### 2.2.2. Digital Surface Loss Analysis (3D Intraoral Scanning)

To objectively quantify enamel wear, a selected subgroup of 40 participants (20 males and 20 females) underwent digital scanning at follow-up. The baseline dental casts were also scanned.

The decision to include only 40 participants (20 males and 20 females) in the digital surface loss analysis was based on logistical and technical considerations. The process of superimposing baseline dental casts with follow-up intraoral scans and performing volumetric enamel loss measurements is highly time-consuming and resource-intensive. The selected sample was designed to be gender-balanced and representative of the broader cohort to allow meaningful correlation analysis between clinical (BEWE) scores and digitally measured enamel loss. This subset approach ensured feasibility without compromising the validity of comparative findings between traditional and digital assessment methods.

Scanning Procedure: Intraoral scans were captured using a MEDIT i700 scanner (Medit Corp., Seoul, Republic of Korea) [17,18]. Superimposition of baseline and follow-up scans was performed using best-fit alignment to detect enamel loss on the buccal and palatal surfaces of anterior teeth. Enamel surface loss >30 µm was defined as clinically significant [1]. For each of the 40 selected participants (20 males and 20 females), intraoral scans were obtained at the 10-year follow-up visit. Baseline records were derived from digitized gypsum casts originally obtained in 2014 and scanned using the same intraoral scanner to ensure consistency. The STL files from baseline and follow-up were aligned using Exocad DentalCAD software (*Galway 3.1*) through a “best-fit” registration protocol. The registration accuracy of the software is within 15 µm, as per the manufacturer′s specifications. Enamel surface loss was calculated through superimposition and 3D subtraction analysis. Surface loss greater than 30 µm was considered clinically significant. The palatal and buccal surfaces of maxillary anterior teeth (central incisors, lateral incisors, and canines) were evaluated, as these are the most erosion-prone regions. This scanning and analysis protocol ensures reproducibility and allows for the quantification of subtle enamel changes that may not be visually apparent.

Digital Outcomes:

- Mean enamel loss per participant (in microns);
- Localization of wear (palatal vs. buccal surfaces);
- Correlation with BEWE score and intraoral scanning.

#### 2.2.3. Demographic Data Collection

Basic demographic variables (age and sex) were collected at baseline. Due to the non-interventional design, no dietary, behavioral, or salivary data were recorded.

#### 2.2.4. Statistical Analysis

Data analysis was conducted using SPSS v 26.0. Descriptive statistics were calculated for demographic data, erosion incidence BEWE distribution, and digital wear measurements. Group comparisons included:

- Chi-square tests for gender differences in erosion incidence;
- Relative risk (RR) with 95% confidence intervals (CI);
- Pearson’s correlation coefficient to evaluate the relationship between clinical scores and 3D enamel loss;
- Statistical significance was set at *p* < 0.05.

## 3. Results

### 3.1. Participant Characteristics

**Figure 2:**
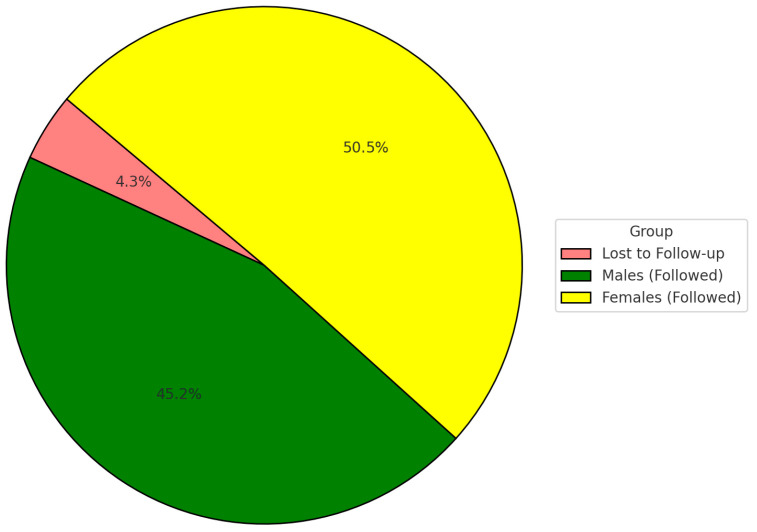
Distribution of total participants.

A total of 540 young adult participants were enrolled at baseline in 2014. At the 2024 follow-up, 517 participants (95.7%) were successfully re-examined and included in the final analysis. The mean age at baseline was 24.7 ± 3.1 years, and the follow-up mean age was 34.7 years. The gender distribution was 244 males (47.2%) and 273 females (52.8%) (Figure 2).

#### Dental Erosion Incidence

**Figure 3:**
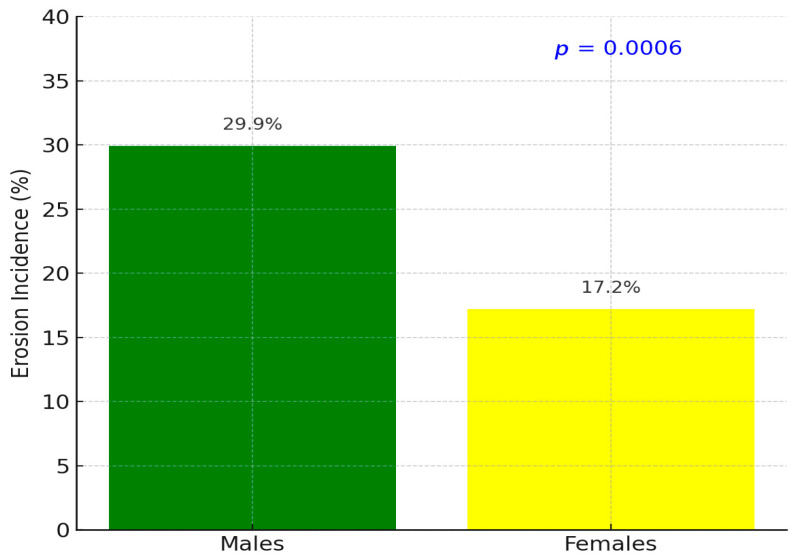
Gender-based incidence (*p* = 0.0006).

Over the 10-year period, 120 of the 517 participants (23.2%) developed dental erosion lesions on at least one tooth surface. Erosive wear incidence differed markedly by gender (Table 1). Males exhibited a higher incidence (73 of 244; 29.9%) compared to females (47 of 273; 17.2%). A Chi-square test confirmed that the incidence of erosion was significantly greater in males, *p* = 0.0006 (statistically significant at *p* < 0.001, Figure 3). The relative risk of developing dental erosion for males versus females was 1.74 (95% confidence interval: 1.26–2.40), indicating that male participants were about 74% more likely to experience new erosive wear over the decade than female participants.

**Figure 4:**
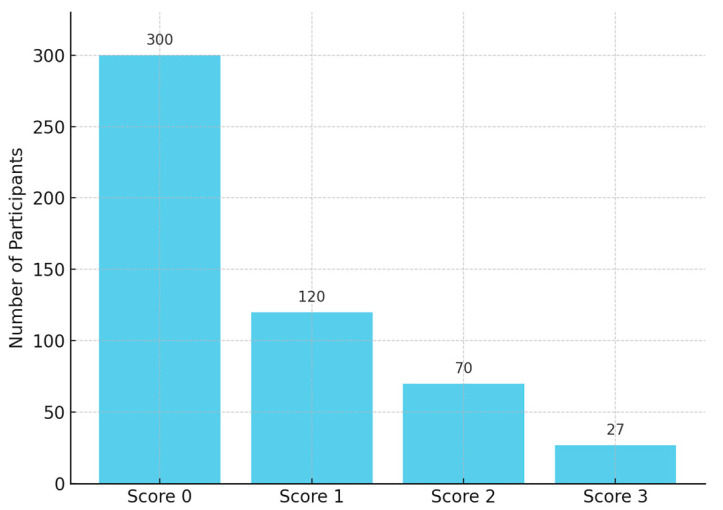
BEWE score distribution (no *p*-value, descriptive). Digital surface loss analysis (*n* = 40).

**Figure 5:**
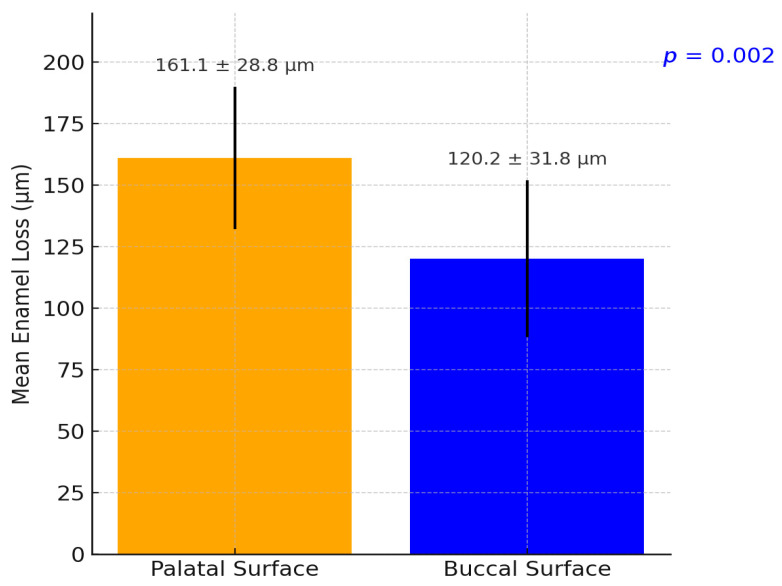
Mean digitally measured enamel surface loss after 10 years (*n* = 40).

**Figure 6:**
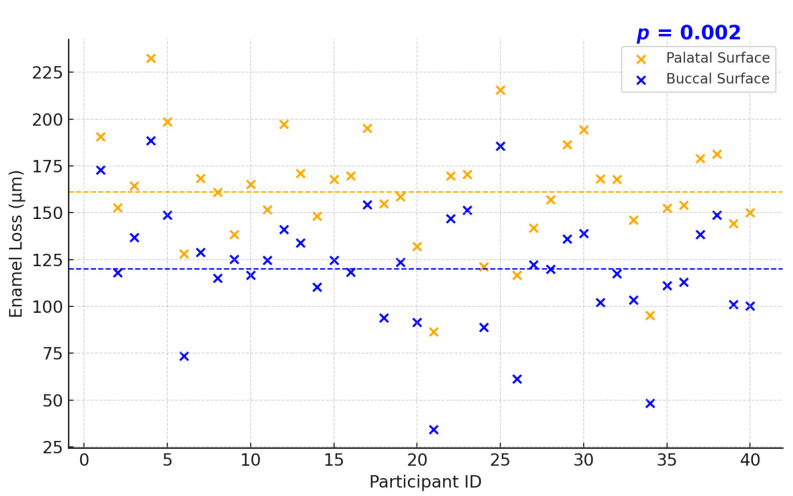
Distribution of mean enamel loss per participant over 10 years in the digital analysis subset (*n* = 40).

The extent of measured enamel loss also correlated positively with the clinical erosion indices recorded at follow-up: participants with higher BEWE scores tended to have greater enamel loss (Figure 4). Three-dimensional volumetric analysis showed a mean enamel thickness loss of approximately 137 ± 79 µm per participant over the decade. Palatal surfaces had significantly greater mean enamel loss than buccal surfaces (mean palatal loss 162 ± 85 µm vs. buccal loss 114 ± 66 µm; *p* = 0.002, paired comparison, Figure 5 and Figure 6). Pearson’s correlation between mean enamel loss and BEWE score was about *r* = 0.58 (*p* < 0.001), indicating a moderate association between the clinically scored wear and actual enamel thickness loss.

**Figure 7:**
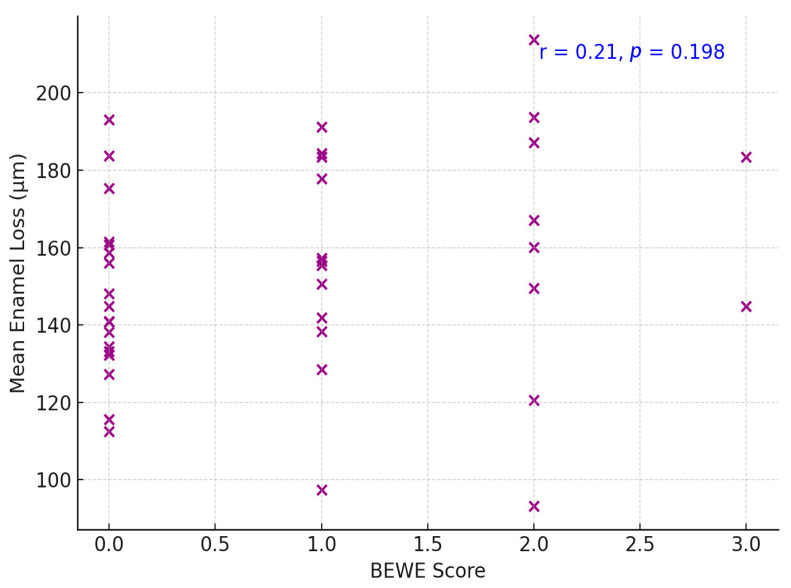
Correlation between BEWE score and digital enamel loss.

Over half the participants exhibited <150 µm of enamel loss on average, whereas a minority (12.5%) experienced ≥250 µm loss. Only one participant exceeded 300 µm of mean loss, indicating that extreme erosive loss was uncommon in this group (Figure 7). The correlation between BEWE scores and digital enamel loss measurements was moderate and statistically significant (*r* = 0.58, *p* < 0.001), indicating a clear relationship between clinical assessments and quantitative digital findings.

### 3.2. Localization of Enamel Loss: Palatal vs. Buccal Surfaces

Analysis of enamel surface loss revealed that the palatal surfaces were predominantly affected in the anterior maxillary region. The greatest loss was observed on the palatal aspects of the central and lateral incisors, followed by the canines. These areas are known to be highly susceptible to intrinsic and extrinsic acid exposure, particularly due to their anatomy and position in the oral cavity. In contrast, the buccal surfaces of these same teeth exhibited significantly less enamel loss. Premolars and molars showed minimal palatal erosion in comparison, with most of the wear in posterior teeth occurring on occlusal or buccal surfaces, if present at all. This distribution aligns with the existing literature, indicating a predilection for palatal erosion in the anterior maxillary teeth due to dietary acids and reflux-related exposure.

## 4. Discussion

Long-term studies show that many young adults develop new erosive tooth wear over a decade. In our Romanian 10-year cohort, we observed a clear increase in both the presence and severity of dental erosion from baseline to follow-up. This trend aligns with international longitudinal data. For example, a Swedish 4-year study in adolescents reported that 76% of initially erosion-free individuals developed erosive lesions over that short period [17]. In Norway, a cohort examined at ages 15 and 18 showed prevalence rising from 51% to 60% (with about 18% of those initially healthy developing erosion in 3 years) [18]. Over six years (ages 15 to 21), prevalence similarly climbed from 57% to 60%, indicating a ~6% incidence of new cases into early adulthood [18]. These findings suggest that the ten-year incidence in young adults internationally may fall in the tens of percentage points, depending on baseline risk and age. Cross-sectional surveys also report a high cumulative prevalence of erosion by the late 20s. In many countries, 30–50% of young adults show signs of erosive wear. Many countries report that roughly 30–50% of young adults exhibit some erosion. In fact, a European multi-center study found over 50% of 18–35-year-olds had at least some dental erosion, and a Swedish study noted prevalence as high as 75% in this age range [18]. Notably, high-income populations often show greater erosion; for instance, surveys in the UK and Scandinavia consistently find over one-third of youths affected [19]. The Romanian cohort’s ten-year results appear to mirror these global patterns, reinforcing that erosive wear is a progressive condition during late adolescence and early adulthood across diverse populations. Factors like increased exposure to dietary acids and changing lifestyles likely drive the upward trend worldwide [20].

### 4.1. Gender Differences in Erosion Risk and Severity

A consistent finding across studies is that males tend to exhibit higher dental erosion prevalence and severity than females in comparable age groups [17]. The Romanian 10-year study reportedly found men experiencing more pronounced progression of erosive lesions than women, which is in line with international data. A large adolescent survey in Stockholm County, Sweden, found dental erosion significantly more prevalent and severe in males than females [17]. Specifically, 15- to 17-year-old boys had higher rates of erosive wear, including severe (dentin-level) lesions, compared to girls [17]. Similarly, a Norwegian study observed that 72% of males (108 of 150) vs. 57% of females (85 of 150) had dental erosion by age 18 (*p* = 0.006) [18]. This male predominance held true for advanced dentin lesions as well (though the gender difference in dentin-level cases was not statistically significant in that sample). Other surveys and reviews concur that male young adults are at higher risk for erosive tooth wear [21,22]. Proposed explanations include behavioral and biological factors: young men may consume acidic beverages (soft drinks, sports drinks, citrus juices) more frequently or in larger volumes, and tend to have riskier dietary habits, leading to greater acid exposure [21,23]. Some authors have also speculated that males’ larger muscle force (heavy chewing/grinding) and possibly thinner enamel in certain cases could exacerbate erosive wear [18]. In contrast, females might benefit from more cautious dietary choices or protective salivary and enamel factors. Indeed, a genetic study suggested females could be less susceptible to erosion, as indicated by the consistently lower prevalence in women [22]. Overall, the evidence strongly supports a gender difference: young males typically present with higher incidence and more severe progression of dental erosion than females, both in Romania and internationally, making gender a recognizable risk indicator in the epidemiology of tooth erosion.

### 4.2. BEWE Index vs. Three-Dimensional Scanning: Diagnostic Accuracy and Correlation

Accurate diagnosis of dental erosion can be challenging. Traditional clinical indices like the Basic Erosive Wear Examination (BEWE) are widely used for scoring erosive wear in different sextants of the mouth. While BEWE is practical and has shown good intra- and inter-examiner reliability in general use [24], it provides only an ordinal grading of surface loss and may underestimate subtle changes. Emerging digital techniques—notably 3D intraoral scanning with model superimposition—allow for quantitative monitoring of tooth surface loss over time. Comparing these methods reveals important differences in sensitivity and accuracy. Research has shown that 3D digital models are especially sensitive in detecting initial erosive wear that might be missed or scored as “sound” in a visual exam [24]. In one validation study, intraoral scanner analysis could reliably detect tissue loss on the order of 50–100 µm, with around 97% accuracy when compared to micro-CT measurements [25]. This means very early enamel erosions (such as beginning cupping or shallow faceting) register on digital overlays even if they are hard to discern clinically. Indeed, when examiners applied the BEWE scoring on digital scans versus directly in the mouth, the digital method often recorded more surfaces with erosive wear or higher BEWE scores than the clinical assessment. For example, Alaraudanjoki et al. found that erosive lesions appeared more extensive on 3D scanned models, and upper posterior tooth surfaces, in particular, tended to be underscored in the clinical exam [24]. In that study, some participants who were deemed erosion-free by clinical BEWE had small lesions visible on the scans; only 6% of subjects were lesion-free on digital models versus 26% clinically [26]. This indicates the higher sensitivity of digital detection in picking up incipient erosion.

However, the concordance (correlation) between clinical scores and digital measurements is only moderate. A recent clinical study on young adults compared BEWE scoring performed intraorally to BEWE on the corresponding 3D scan models. The agreement was fair-to-moderate (weighted kappa in the range 0.4–0.6), and it was poorest for molar occlusal surfaces [26]. Interestingly, examiners often assigned higher scores on the digital models for the same tooth than they did in person, especially for the posterior teeth [26]. The discrepancy may arise because, on a screen, one can enlarge and inspect the 3D model closely from all angles, detecting even tiny “cuppings” or wear facets, whereas clinically those might be overlooked or not judged as significant under standard lighting [26]. On the other hand, digital models lack tactile feedback and subtle color changes (like enamel translucency or dentin shining through) that clinicians use to gauge depth of erosion [26]. For instance, BEWE scoring criteria consider mainly the area of surface involved, not the depth; a very small but deep erosion into dentin still only scores 1 on BEWE if it is under 50% of the surface [26]. An examiner might intuitively rate such a lesion higher in severity when seeing it in person (due to visible dentin), potentially causing inconsistency with the model-based score. Thus, while digital scanning offers superior precision and the ability to monitor volumetric loss over time, it does not perfectly mirror clinical index assessments. Researchers conclude that 3D scans are an excellent adjunct for early detection and serial monitoring of erosive wear—one study noted no patient was entirely erosion-free when combining clinical and digital detection [26]—but the BEWE index remains a convenient chairside tool for screening. The two approaches are moderately correlated; they generally identify the same individuals with heavy wear but may differ in grading subtle lesions. In practice, using both methods in complement can improve diagnostic accuracy: the BEWE can flag patients with erosive wear risk, and intraoral scanning can document baseline lesions and quantify small changes at recalls. As digital technology advances, we may see improved software that correlates better with clinical indices or even new indices tailored for digital model analysis [26].

Also, other authors suggested that etiological factors of erosive tooth wear should be considered and scored for differential diagnosis and risk assessment [27,28].

The correlation observed between BEWE scores and digitally measured enamel loss (*r* = 0.58) reflects a moderate association, indicating that while the two methods generally align in identifying cases with more advanced wear, they are not directly interchangeable. BEWE is a visual, ordinal index that captures the most severely affected surface in each sextant, while digital surface analysis provides a quantitative, three-dimensional evaluation of enamel volume loss across specific regions [1]. As such, discrepancies between the two are expected, especially in early or localized lesions that may fall below the BEWE detection threshold but are measurable on 3D scans. These findings suggest that BEWE and digital scanning provide complementary—rather than equivalent—diagnostic insights. The use of both tools in combination may enhance erosion monitoring, especially in longitudinal studies and individualized preventive care [27].

Although the present study did not include structured follow-up on behavioral or lifestyle changes, it is plausible that shifts in diet, oral hygiene, or general health habits over the ten-year period contributed to the development of dental erosion in affected individuals. Previous studies have shown that increased consumption of acidic beverages, changes in occupational stress, dietary patterns, and even fitness-related supplement use can elevate the risk of erosive wear. In the absence of longitudinal behavioral data, these contributing factors remain speculative within our cohort. Nonetheless, the observed gender differences and incidence trends suggest that certain routine-related variables may have influenced erosion onset. Future studies should incorporate detailed questionnaires and clinical interviews to track changes in patient routines and correlate them with erosion progression for a more comprehensive understanding of causality and risk modification [2,3].

This study has several limitations that should be acknowledged. First, the digital surface loss analysis was conducted on a relatively small subsample of 40 participants, which may limit the generalizability of the quantitative findings. Although this subset was balanced by gender and representative of the larger cohort, the limited number reduces statistical power for subgroup analyses. Second, no intermediate assessments were conducted during the 10-year follow-up period. As a result, the exact timeline and progression pattern of enamel loss remain unknown. Third, no data were collected regarding dietary acid exposure, fluoride usage, or behavioral habits such as frequency of acidic beverage consumption or oral hygiene routines—all of which are known contributors to dental erosion. The absence of these risk factor assessments restricts our ability to analyze etiological influences or protective behaviors. Finally, the study population consisted exclusively of individuals with no clinical signs of erosion at baseline (BEWE = 0). While this allows for clear incidence tracking, it may not reflect erosion patterns in populations with pre-existing wear, limiting the external validity of our findings [1,28].

Both the BEWE index and digital intraoral scanning have inherent strengths and limitations that can influence diagnostic outcomes. BEWE, as a visual index based on surface area involvement, may underestimate early or subtle lesions—particularly those confined to non-prominent surfaces or lacking significant area involvement. In contrast, digital scanning, with its high-resolution surface mapping and superimposition capabilities, can detect minute changes in enamel thickness that may not be clinically apparent. While this enhances sensitivity, it also introduces the possibility of overestimating clinical significance, especially when early wear does not yet warrant intervention. Additionally, the feasibility of widespread digital scanning use in epidemiological or routine dental settings remains limited by factors such as cost, required operator training, time consumption, and software access. These logistical challenges currently restrict the integration of 3D scanning into general practice, despite its potential as a research and monitoring tool. Therefore, both approaches should be viewed as complementary, with digital methods best suited for detailed longitudinal evaluation and BEWE serving as a practical, quick screening tool in daily practice [1].

## 5. Conclusions

1. Longitudinal Erosion Rates: The Romanian 10-year study confirms that dental erosion accumulates appreciably in young adults over time, in line with global data. International longitudinal studies report a substantial 5–10 year incidence of erosive wear in this age group (on the order of tens of percent), with prevalence often rising from around one-quarter in the late teens to over one-third or more by the late twenties.
2. Gender Differences: Males consistently show higher susceptibility to dental erosion than females. Romanian findings and worldwide evidence concur that young men experience higher incidence and greater severity of erosive tooth wear than women of similar age. Behavioral factors (e.g., more acidic drink consumption) and possible biological differences likely contribute to this male predominance.
3. Diagnostic Methodologies: There is a meaningful difference between clinical index scoring and digital detection of erosion. The BEWE index is practical for in vivo assessment but may underestimate early or localized erosions, whereas 3D intraoral scanning can detect and measure very initial enamel wear with high sensitivity. Studies show only moderate agreement between BEWE scores and digital measurements, implying that combining traditional and digital methods provides a more complete picture of erosive wear status.
4. In summary, 3D scanning offers superior accuracy in quantifying erosion progression, while BEWE remains a valuable, validated tool for quick clinical evaluation—the two should be viewed as complementary in monitoring dental erosion.
5. Additionally, advancements in 3D-printing technologies offer promising opportunities for the digital rehabilitation of dentitions affected by advanced erosive tooth wear, supporting the integration of additive manufacturing in restorative workflows. Furthermore, 3D-printing technologies may play an important role in the digital rehabilitation of erosively worn dentitions, offering additive manufacturing solutions for customized restorative treatment [28].

## Figures and Tables

**Table 1:** Ten-year incidence of dental erosion by gender (*n* = 517).

Gender	Participants (*n*)	With Erosion (*n* %)	Without Erosion (*n* %)
Males	244	73 (29.9%)	171 (70.1%)
Females	273	47 (17.2%)	226 (82.8%)
Total	517	120 (23.2%)	397 (76.8%)

## Data Availability

The data presented in this study are available on request from the corresponding author.

## References

[1] Schlueter N., Luka B. (2018). Erosive tooth wear—A review on global prevalence and on its prevalence in risk groups. Br. Dent. J..

[2] Avila V., Betlrán E.O., Cortés A., Usuga-Vacca M., Castellanos Parras J.E., Diaz-Baez D., Martignon S. (2024). Prevalence of erosive tooth wear and associated risk factors in Colombian adolescents. Braz. Oral Res..

[3] de la Parte-Serna A.C., Monticelli F., Pradas F., Lecina M., García-Giménez A. (2025). Gender-Based Analysis of Oral Health Outcomes Among Elite Athletes. Sports.

[4] Quinchiguano Caraguay M.A., Amoroso Calle E.E., Idrovo Tinta T.S., Gil Pozo J.A. (2023). Non-carious cervical lesions (NCCL): A review of the literature. RSD [Internet].

[5] Hasselkvist A., Arnrup K. (2021). Prevalence and progression of erosive tooth wear among children and adolescents in a Swedish county, as diagnosed by general practitioners during routine dental practice. Heliyon.

[6] Chan A.S., Tran T.T.K., Hsu Y.H., Liu S.Y.S., Kroon J. (2020). A systematic review of dietary acids and habits on dental erosion in adolescents. Int. J. Paediatr. Dent..

[7] Kong W., Ma H., Qiao F., Xiao M., Wang L., Zhou L., Chen Y., Liu J., Wang Y., Wu L. (2024). Risk factors for noncarious cervical lesions: A case-control study. J. Oral Rehabil..

[8] Methuen M., Kangasmaa H., Alaraudanjoki V.K., Suominen A.L., Anttonen V., Vähänikkilä H., Karjalainen P., Väistö J., Lakka T., Laitala M.L. (2022). Prevalence of Erosive Tooth Wear and Associated Dietary Factors among a Group of Finnish Adolescents. Caries Res..

[9] Jász M., Szőke J. (2022). Dental Erosion and Its Relation to Potential Influencing Factors among 12-year-old Hungarian Schoolchildren. Oral Health Prev. Dent..

[10] Piórecka B., Jamka-Kasprzyk M., Niedźwiadek A., Jagielski P., Jurczak A. (2023). Fluid Intake and the Occurrence of Erosive Tooth Wear in a Group of Healthy and Disabled Children from the Małopolska Region (Poland). Int. J. Environ. Res. Public Health.

[11] West N.X., Davies M., Sculean A., Jepsen S., Faria-Almeida R., Harding M., Graziani F., Newcombe R.G., Creeth J.E., Herrera D. (2024). Prevalence of dentine hypersensitivity, erosive tooth wear, gingival recession and periodontal health in seven European countries. J. Dent..

[12] Inchingolo F., Dipalma G., Azzollini D., Trilli I., Carpentiere V., Hazballa D., Bordea I.R., Palermo A., Inchingolo A.D., Inchingolo A.M. (2023). Advances in Preventive and Therapeutic Approaches for Dental Erosion: A Systematic Review. Dent. J..

[13] Tapalaga G., Bumbu B.A., Reddy S.R., Vutukuru S.D., Nalla A., Bratosin F., Fericean R.M., Dumitru C., Crisan D.C., Nicolae N. (2023). The Impact of Prenatal Vitamin D on Enamel Defects and Tooth Erosion: A Systematic Review. Nutrients.

[14] Aránguiz V., Lara J.S., Marró M.L., O’Toole S., Ramírez V., Bartlett D. (2020). Recommendations and guidelines for dentists using the basic erosive wear examination index (BEWE). Br. Dent. J..

[15] Bartlett D.W., Ganss C., Lussi A. (2008). Basic Erosive Wear Examination (BEWE): A new scoring system for scientific and clinical needs. Clin. Oral Investig..

[16] O’Toole S., Bartlett D., Keeling A., McBride J., Bernabe E., Crins L., Loomans B. (2020). Influence of Scanner Precision and Analysis Software in Quantifying Three-Dimensional Intraoral Changes: Two-Factor Factorial Experimental Design. J. Med. Internet Res..

[17] Skalsky Jarkander M., Grindefjord M., Carlstedt K. (2018). Dental erosion: Prevalence and risk factors among a group of adolescents in Stockholm County. Eur. Arch. Paediatr. Dent..

[18] Stenhagen K.R., Berntsen I., Ødegaard M., Mulic A., Tveit A.B. (2017). Has the prevalence and severity of dental erosion in Norwegian adolescents changed over 30 years?. Eur. J. Paediatr. Dent..

[19] Hasselkvist A., Johansson A., Johansson A.K. (2016). A 4-year longitudinal study of progression of dental erosion in Swedish adolescents. J. Dent..

[20] Bratu D.C., Mihali S.G., Popa G., Dragoş B., Bratu R.C., Matichescu A., Loloș D., Boloș O. (2024). The Prevalence of Dental Erosion in Young Adults—A Quantitative Approach. Med. Evol..

[21] Søvik J.B., Skudutyte-Rysstad R., Tveit A.B., Sandvik L., Mulic A. (2015). Sour Sweets and Acidic Beverage Consumption Are Risk Indicators for Dental Erosion. Caries Res..

[22] Uhlen M.M., Stenhagen K.R., Dizak P.M., Holme B., Mulic A., Tveit A.B., Vieira A.R. (2016). Genetic variation may explain why females are less susceptible to dental erosion. Eur. J. Oral Sci..

[23] Avila V., Beltrán E.O., Cortés A., Usuga-Vacca M., Castellanos Parras J.E., Díaz-Báez D., Martignon S. (2024). Prevalence of erosive tooth wear and associated risk factors in Colombian adolescents. Braz. Oral Res..

[24] Alaraudanjoki V., Saarela H., Pesonen R., Laitala M.L., Kiviahde H., Tjäderhane L., Lussi A., Pesonen P., Anttonen V. (2017). Is a Basic Erosive Wear Examination (BEWE) reliable for recording erosive tooth wear on 3D models?. J. Dent..

[25] Mitrirattanakul S., Neoh S.P., Chalarmchaichaloenkit J., Limthanabodi C., Trerayapiwat C., Pipatpajong N., Taechushong N., Chintavalakorn R. (2023). Accuracy of the Intraoral Scanner for Detection of Tooth Wear. Int. Dent. J..

[26] Al-Seelawi Z., Hermann N.V., Peutzfeldt A., Baram S., Bakke M., Sonnesen L., Tsakanikou A., Rahiotis C., Benetti A.R. (2024). Clinical and digital assessment of tooth wear. Sci. Rep..

[27] Margaritis V., Mamai-Homata E., Koletsi-Kounari H. (2011). Novel methods of balancing covariates for the assessment of dental erosion: A contribution to validation of a synthetic scoring system for erosive wear. J. Dent..

[28] Patrício T., Domingos M., Gloria A., Bartolo P.J. (2012). Advanced Additive Manufacturing Technologies for the Production of Oral and Maxillofacial Implants. Materials.

